# PM_2.5_ concentration prediction based on EEMD-ALSTM

**DOI:** 10.1038/s41598-024-63620-9

**Published:** 2024-06-02

**Authors:** Zuhan Liu, Dong Ji, Lili Wang

**Affiliations:** 1 School of Information Engineering, Nanchang Institute of Technology, Nanchang, 330099 China; 2 College of Science, Nanchang Institute of Technology, Nanchang, 330099 China

**Keywords:** Ensemble empirical mode decomposition, Long short-term memory network, Attention mechanism, Air pollution, PM_2.5_, Environmental sciences, Mathematics and computing

## Abstract

The concentration prediction of PM_2.5_ plays a vital role in controlling the air and improving the environment. This paper proposes a prediction model (namely EEMD-ALSTM) based on Ensemble Empirical Mode Decomposition (EEMD), Attention Mechanism and Long Short-Term Memory network (LSTM). Through the combination of decomposition and LSTM, attention mechanism is introduced to realize the prediction of PM_2.5_ concentration. The advantage of EEMD-ALSTM model is that it decomposes and combines the original data using the method of ensemble empirical mode decomposition, reduces the high nonlinearity of the original data, and Specially reintroduction the attention mechanism, which enhances the extraction and retention of data features by the model. Through experimental comparison, it was found that the EEMD-ALSTM model reduced its MAE and RMSE by about 15% while maintaining the same R^2^ correlation coefficient, and the stability of the model in the prediction process was also improved significantly.

With the rapid development of Chinas economy and the acceleration of urbanization, environmental pollution problems are becoming increasingly serious, especially the haze pollution mainly caused by PM_2.5_. This type of pollution not only puts pressure on the urban atmospheric environment, but also threatens public health and affects economic sustainability. Therefore, effective prediction and control of PM_2.5_ concentration have become crucial. Accurate prediction helps the government to implement early warning and response measures in a timely manner, reducing pollutant emissions; Assist the public in taking advance health protection measures; And provide a basis for scientific research, understand the causes and evolution laws of haze, and formulate effective control strategies^1,2^. In summary, haze prediction is a key issue involving public health, economic development, and scientific research.

At present, the main prediction methods for PM_2.5_ concentration are mainly divided into physics-driven and data-driven method. Specifically, physics-driven models are mainly established through the physical and chemical factors and formation processes that affect PM_2.5_. Among them, the community multi-scale air quality prediction model proposed by Byun and Schere^3^ is a representative of physical driven methods^4^. Data-driven models are mainly based on time series analysis and machine learning, with traditional machine learning methods represented by Support Vector Machines (SVM). For example, a hybrid multitasking model based on SVM can effectively overcome spatiotemporal relationships. These traditional machine learning methods have a certain ability to handle nonlinear data, but for processing large amounts of data information, machine learning methods cannot gain a deeper understanding of the information contained in the data^5^.

However, with the rapid development of deep learning, predictive models based on deep learning have attracted more and more attention, and the most prominent feature of deep learning is its deeper network structure^6^. The Recurrent Neural Networks (RNN) is a type of neural network that has outstanding advantages in processing time series data and predicting sequence data during the development of deep learning technology^7,8^. As the most commonly used variant of RNN, LSTM solves the problem of long-range dependence by adding gate and memory units and can preserve the temporal information contained in the data. In recent years, many new models have been constructed based on LSTM and have demonstrated excellent performance in corresponding tasks^9,10^; for instance, the convolutional neural network, the bidirectional LSTM deep learning model CR-LSTM and RBF-LSTM model combined with RBF are improved based on its excellent performance in natural language processing problems^11–14^. Meanwhile, attention mechanism serves as a special structure embedded in deep learning models. It can further extract data features from the network model and enhance the effective features transmitted to the next level in the network^13,15^. Therefore, the addition of attention has been proven to have a significant improvement in the accuracy of prediction results in many prediction models.

The process of data is also an important aspect that affects the quality of machine learning results. The method of decomposition and combination is mainly used for data signal processing. In natural language processing tasks, most of the data is highly non-linear, so how to reduce the dimensionality of the highly non-linear data information is one of the important issues in natural language processing tasks. Empirical Mode Decomposition (EMD) proposed by Huang et al. is a data processing method that decomposes signals into different time and frequency domains, and then reduces nonlinearity in the signal^16,17^. However, due to the modal aliasing problem of EMD, some scholars try to make predictions with EEMD to process data^18,19^. In addition, Yang et al. used decomposition methods such as EEMD to reduce the nonlinearity of the data by using signal decomposition to deepen their understanding of the data^20^. Zhu et al. demonstrated through experiments that the use of attention mechanisms can enhance the important role of predictive models in extracting data features and deepening understanding of data^21^. The above proves the feasibility of EEMD and attention mechanism in deeply understanding data from two aspects: input and extraction.

At present, the numerous models mentioned above extremely rely on multiple influencing factors for prediction^20,22^, which predict various factors that affect air quality or PM_2.5_. The advantage of these models is that these prediction methods can jointly influence the direction of prediction based on these different influencing factors, but this is also a disadvantage of this model, these large and diverse experimental data require a lot of equipment to collect. To address this issue, this article proposes to use a single data in the dataset for long-term prediction of PM_2.5_. The method of EEMD and attention mechanism first decomposes the original signal to reduce the dimensionality of the original data features, and then adds attention mechanism to enhance the models method of extracting and focusing on data features for prediction.

## Materials and methods

### Materials

This article takes changes of the PM_2.5_ in Beijing as an example, using a dataset from the machine learning library of the University of California. The dataset includes 43,824 h of air data from January 31, 2010 to December 31, 2014, which includes real-time PM_2.5_ concentration, dew point, temperature, wind direction, air pressure, and other items per hour of Beijing. This article mainly uses real-time data of PM_2.5_ for experiments. The data can be found on the website: https://archive.ics.uci.edu/dataset/381/beijing+pm2+5+data.

Among them, the average concentration of PM_2.5_ is 71.099 μg/m^3^, the minimum value is 2.000 μg/m^3^, and the maximum value reaches 882.000 μg/m^3^. For missing data, they are deleted, and then the remaining data are going to be reordered by date. Finally, the processed data will write into a new table.

### Methods

In this section, we will introduce the details of EEMD, attention mechanism and LSTM. And based on the above three models, a EEMD-ALSTM model is constructed. The goal of the model is to predict the actual PM_2.5_ concentration after 24 h. Also, it will predict the sub signals generated in the model simultaneously. Therefore, it is a class multi-step prediction model.

1. *EEMD* The EEMD method is a locally adaptive time series analysis technique developed in recent years, which is a new nonlinear and non-stationary time series analysis method based on the empirical model decomposition (EMD) method^23^. The essence of EMD is to decompose nonlinear signals into intrinsic mode signals at different time scales using both time-domain and frequency-domain processing methods. The decomposed signal has local signal characteristics of the original signal at the same time scale^24^. That is to decompose a signal into multiple signals containing the intrinsic modes of the original signal. However, the EMD method not only incorporates features from other time scales during the decomposition process, resulting in modal aliasing, but also lacks the standard to stop iteration during the iteration process^25^. The advantage of EMD is its strong adaptability, which can decompose signals with different cut-off frequencies and bandwidths based on the original signal. However, it also expresses the properties of the original signal in each component. The disadvantage is that different signals at the same time scale have the phenomenon of similar scale signals and endpoint effects. The EEMD just makes up for these shortcomings. The model starts with adding noise and increases the number of zeros in the original signal to assist in signal processing^23^. It first add positive Gaussian white noise to the original signal, treat the added signal as a whole, and then perform EMD decomposition on the signal to obtain each modal component IMFs. Then, repeat the above steps to obtain the IMFs component after each EMD decomposition. As a note, whether the decomposed IMF components belong to pure noise or physically significant components of the original sequence can be determined through significance testing. Its properties can be determined by energy spectral density period distribution of each IMF component and then IMFs are selected you want. The specific process of EEMD decomposition is shown in Fig. 1.

**Figure 1:**
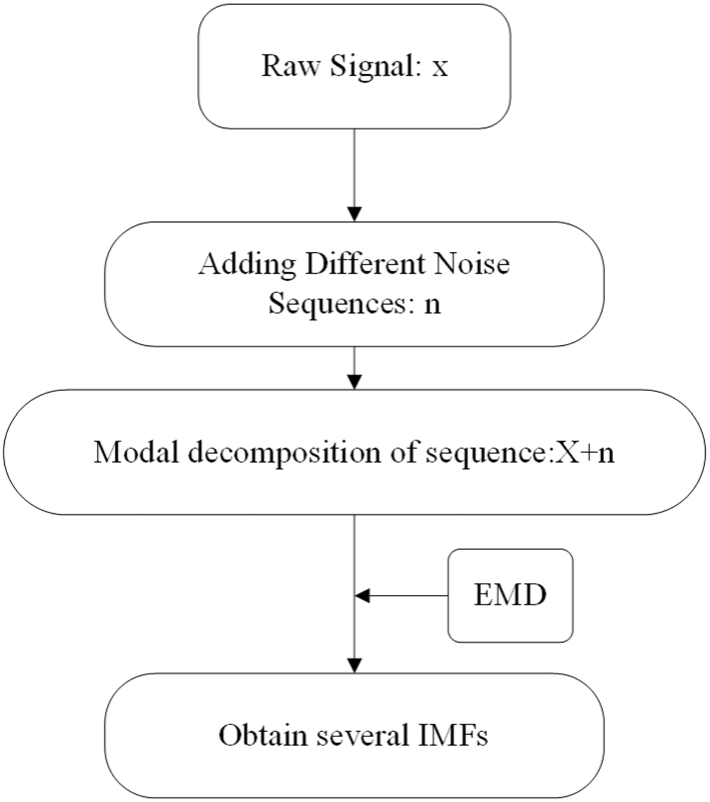
The process of EEMD decomposition.
2. *LSTM* RNN is a type of neural network model in the field of deep learning, which consists of a series of recurrent self-connecting structures^8,26^, as shown in Fig. 2. Its main drawback is the long-range dependence problem caused by gradient explosion and gradient disappearance^27,28^. Moreover, there will be a large amount of continuous data in the calculation process of backpropagation algorithm over time. And if the time interval is too small, the gradient will disappear, and if the interval is too large, the gradient explosion will occur, leading to the instability of the entire neural network.

**Figure 2:**
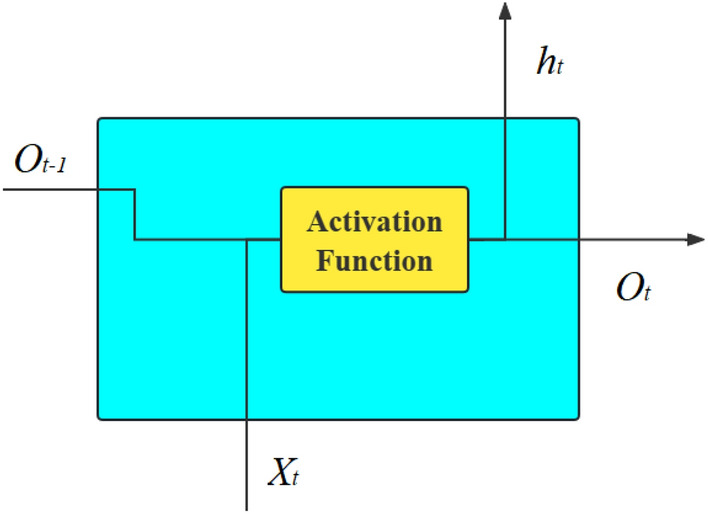
Recurrent neural network.
  The LSTM is an improved neural network based on RNN that have insufficient ability to solve long-term dependency problems, as shown in Fig. 3.

**Figure 3:**
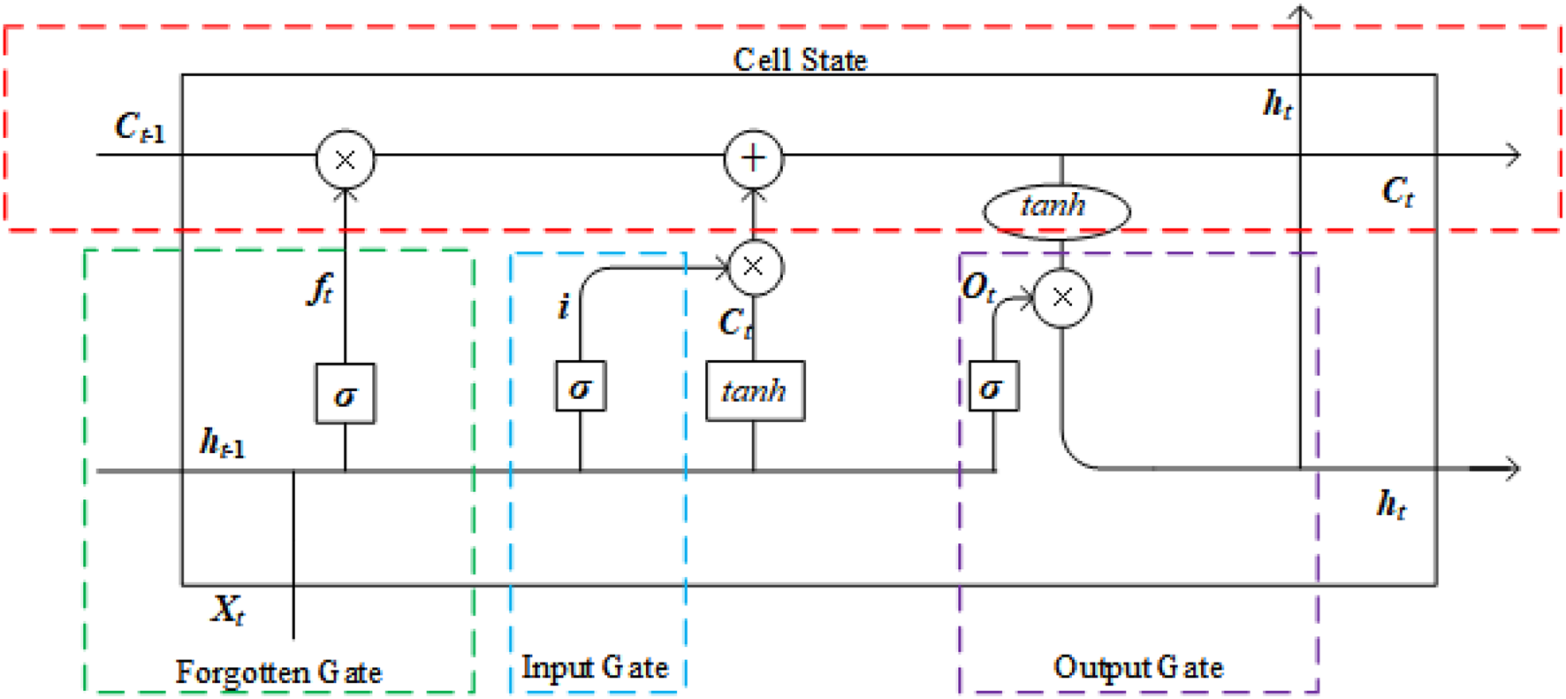
The structure of LSTM.
3. *Attention Mechanism* Attention mechanism is a way of simulating the processing of information by the human brain, which has gradually been used in the field of deep learning in recent years. Drawing on the attention mechanism of the human brain, the model is focused on more important feature information, and a larger weight ratio is assigned to it during model training. For relatively unimportant information, smaller weights are assigned to reduce sensitivity to secondary information, thereby improving the models data processing ability when the models computing power is limited^29^. And in LSTM, attention mechanism weighting based on different features in temporal information can improve the correlation of neural network predictions.
  Figure 4 illustrates the essence of attention mechanism. It adds a linear transformation node inside the neural network, pays attention to the data features of the input neural network, and then assigns different weights based on the distribution of attention. Its formula as
  1 $${\text{att}}(X,q) = \sum\limits_{i = 1}^{N} {\alpha_{i} x_{i} }$$
  where *x*, *q*, *α*_*i*_ and *x*_*i*_ represent the input sequence, the feature, the distribution of attention and the number *i* information in the sequence respectively.

**Figure 4:**
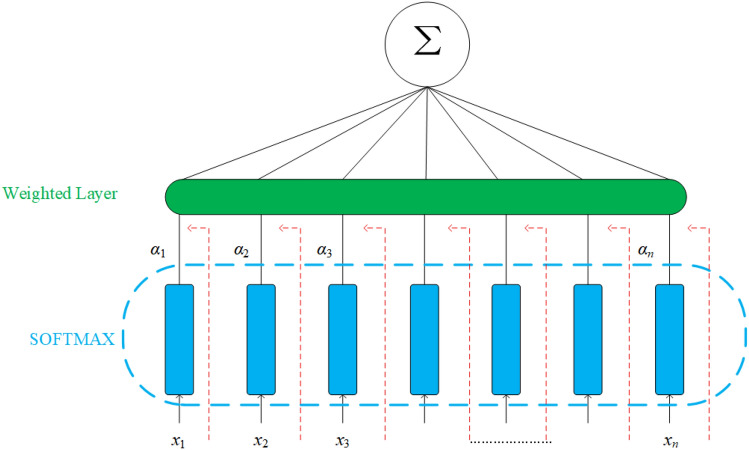
Attention mechanism.
4. *ALSTM* The model enhances the LSTM networks ability to process sequential data by introducing attention mechanisms. In ALSTM, a memory system is usually designed that can store and update information, and uses attention mechanisms to focus on important parts of data in real time. Compared to traditional LSTM, the main structural difference of ALSTM is that it adds an attention layer in the network. This additional layer enables the model to comprehensively consider and focus on key time steps in the input sequence before making predictions. The specific process is shown in the Fig. 5.

**Figure 5:**
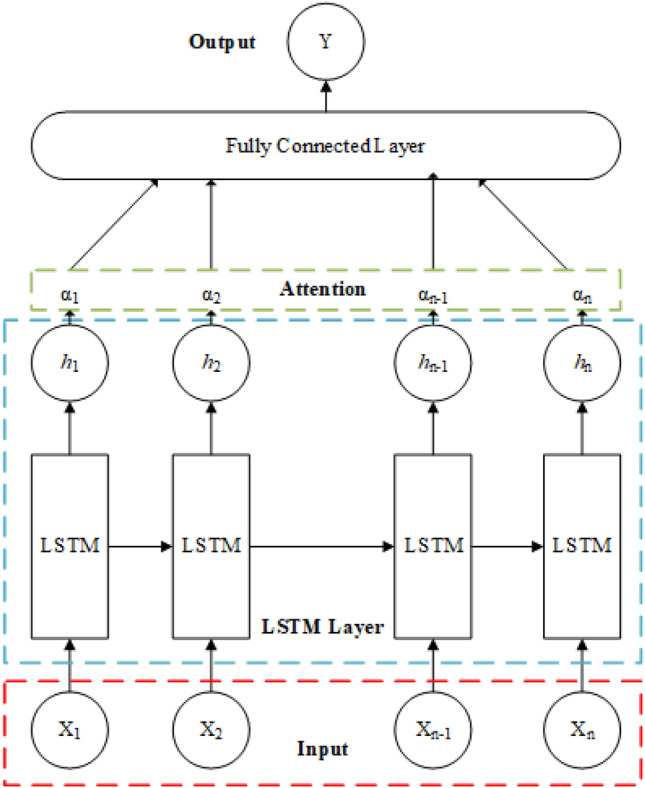
Structural diagram of ALSTM model.
5. *EEMD-ALSTM model* The model is constructed based on the above three basic models as shown in Fig. 6. It consists of three main components, namely EEMD for temporal data, LSTM neural network and attention mechanism. On the left side of Fig. 6, the downloaded dataset is organized into a data table that is only related to PM_2.5_ concentration and then transmitted to the network. During the initial processing of these data, the data containing PM_2.5_ is organized and written into a new table for future use. Then re-read the new data table, convert it into the model, and decompose it through EEMD to decompose the highly nonlinear original signal of PM_2.5_ concentration into a simple oscillation signal at a local time scale. Summarize the decomposed modal signals and remaining signals into a table, and then perform dataset segmentation, data type conversion and data format conversion on each modal signal. Using 24 time points of PM_2.5_ concentration data as input feature vectors, predict each signal separately. It is worth emphasizing that in the prediction process, in addition to using the LSTM model for overall prediction, attention mechanisms are also used to enhance feature vectors. Here, we will connect the hidden layer *h*_*t*_ of LSTM with the attention layer *x*_*t*._ This increase in attention can make the prediction model more closely connect the data information and changes of the upper and lower layers. Finally, the predictions for each signal are combined to form the final output.

**Figure 6:**
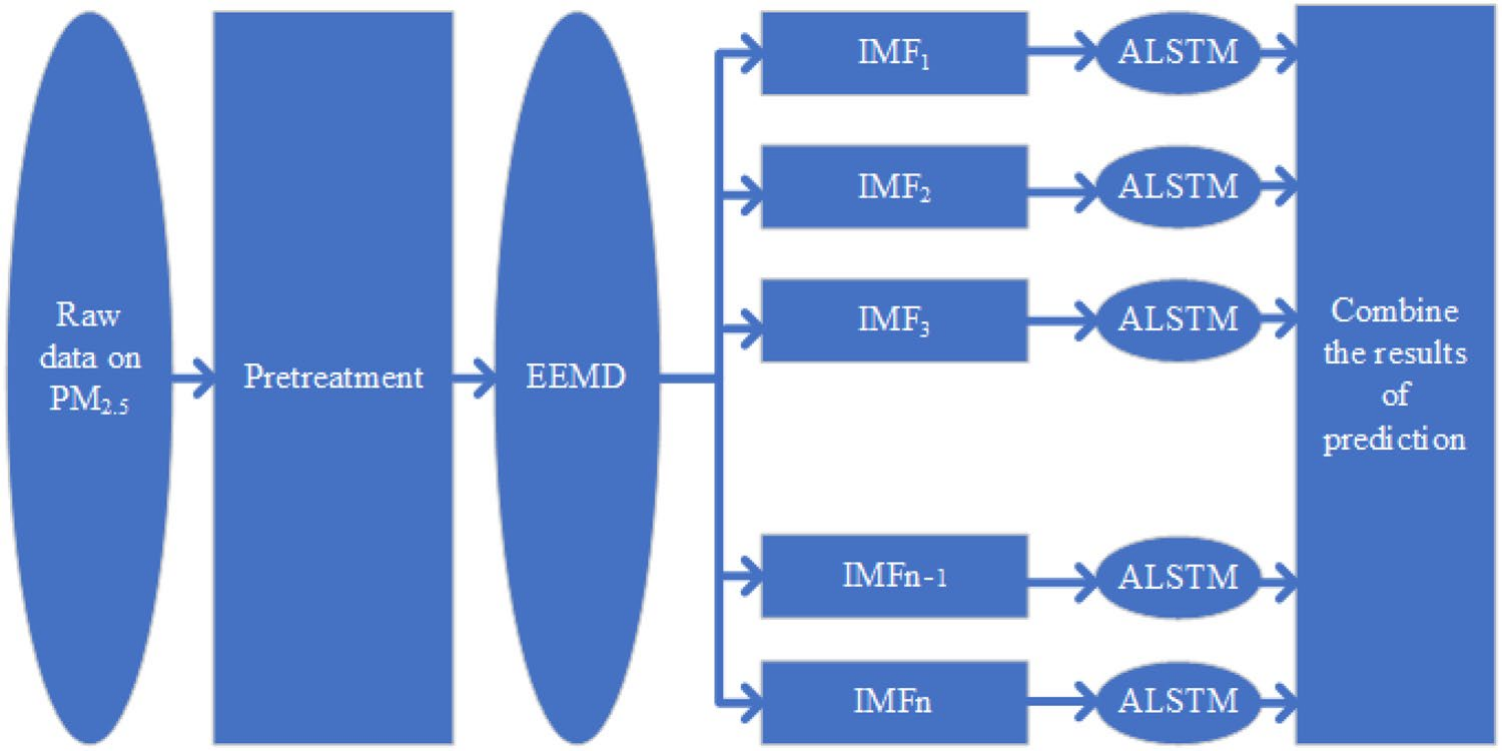
The EEMD-ALSTM model framework.
6. *Evaluating Indicator* In order to evaluate and compare the predictive models involved in this experiment, the R^2^ coefficient of determination (R-Square), root mean square error (RMSE) and mean absolute error (MAE) were used to evaluate the predictive accuracy of the models. The calculation formula for evaluation indicators is as follows:
  2 $${\text{R}}^{2} = 1 - \frac{{\sum\limits_{h = 1}^{H} {\left( {Y_{h} - g(X_{h} )} \right)^{2} } }}{{\sum\limits_{h = 1}^{H} {\left( {\overline{{Y_{h} }} - g(X_{h} )} \right)} }}$$
  3 $${\text{RMSE}} = \sqrt {\frac{1}{H}\sum\limits_{h = 1}^{H} {\left( {Y_{h} - g(X_{h} )} \right)^{2} } }$$
  4 $$MAE = \frac{1}{H}\sum\limits_{h = 1}^{H} {\left| {Y_{h} - g(X_{h} )} \right|}$$

## Results

In this work, we first compared the EEMD-ALSTM model with other models and predicted the PM_2.5_ content one hour later. This article only displays the predicted results of the test set of these four different models. Set the prediction results from the first hour to the 24th hour as case No. 1, then set the prediction results from the second hour to the 25th hour as case No. 2, and so on.

**Figure 7:**
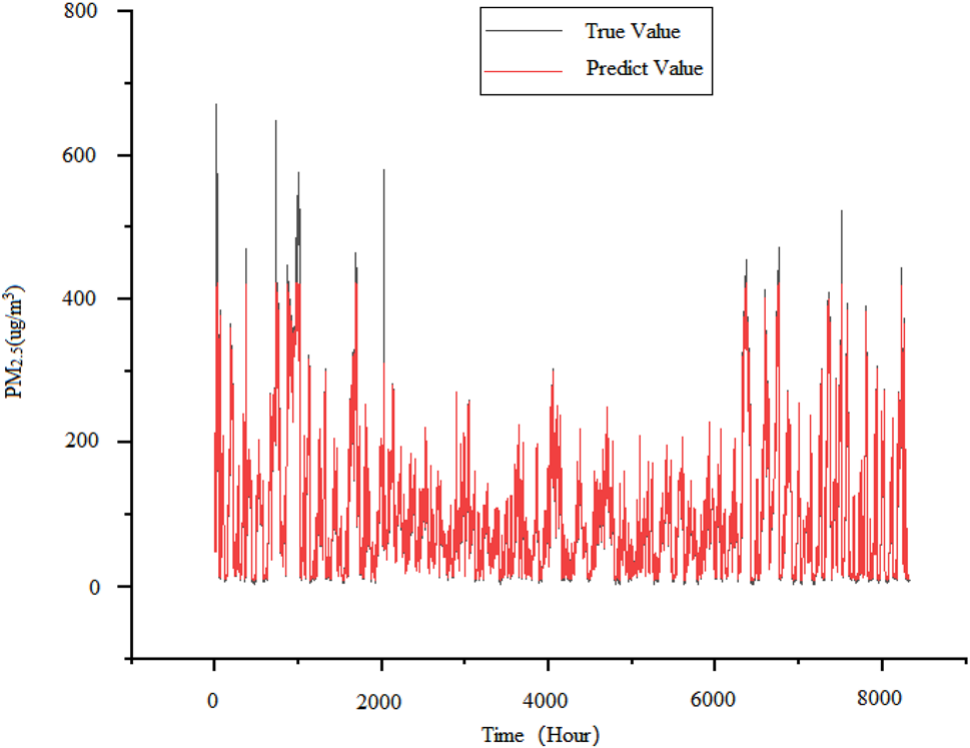
Predicted demerits of SVR model.

SVR is a commonly used regression problem model used to solve time series problems^30–32^. Due to the input sequence format requirements of the SVR model, we changed the format of the time series data to one-dimensional and used “RBF” as the kernel function to call the SVM toolbox from sklearn for experiments. Therefore, the horizontal axis of the predicted results using support vector regression is displayed as “Time (Hour)”. The test set results obtained from the experiment using the above dataset are shown in the Fig. 7. The advantage of SVR model lies in its small size, ability to handle high-dimensional data, and ability to handle linear and nonlinear problems. However, the disadvantage of the SVR model is that it does not support parallel computing, so it may encounter performance bottlenecks when processing.large-scale data. And SVR belongs to machine learning models. For large-scale datasets, SVR models have high computational complexity and long training time; During the training process, it is sensitive to parameter adjustment and outliers in the data, as shown in Fig. 7. When approaching the peak of PM_2.5_ concentration, the predicted curve cannot fully fit the true curve. Therefore, it is necessary to choose different kernel functions and regularization to prevent.

The first step in establishing an LSTM model is to determine the input layer, intermediate layer, and output layer. Among them, the input layer receives the raw data in the format of 32 neurons and input vector [24:1], and two LSTM layers with 32 neurons are set in the middle to extract features and learn. Finally, a fully connected layer is used as the output layer to predict the learned features. Compared to regular RNNs, LSTM adds an additional memory module that can better learn and store long-term information. The test set results obtained from the experiment using LSTM prediction model and the aforementioned dataset are shown in Fig. 7.

**Figure 8:**
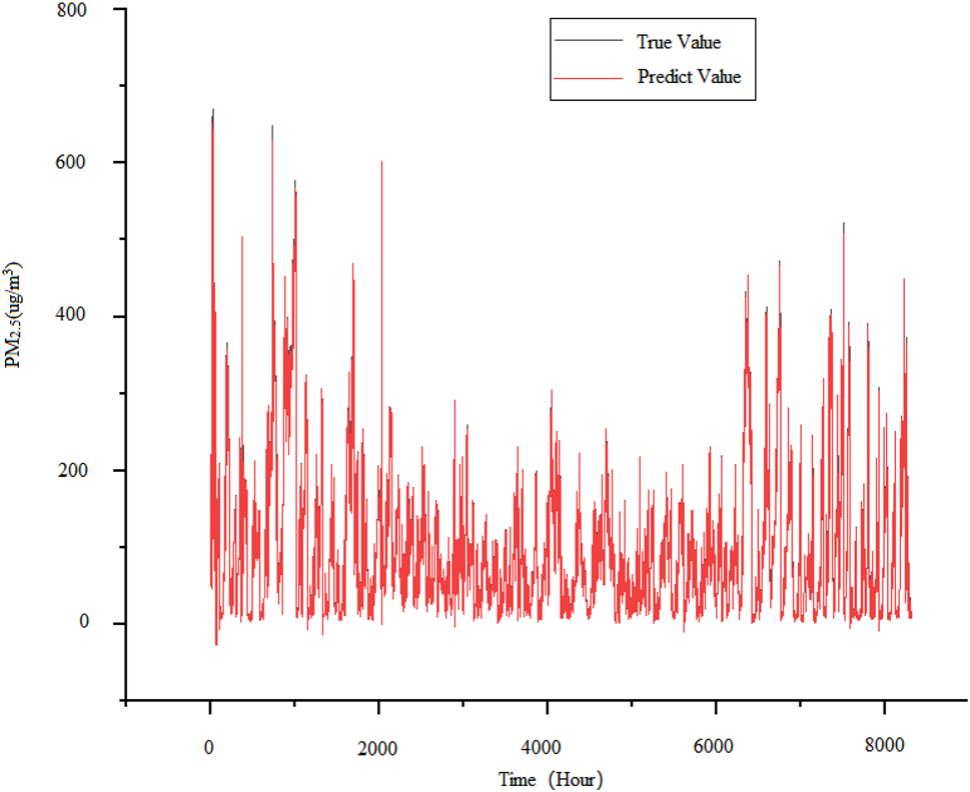
Predicted demerits of LSTM model.

In the Fig. 8, we can clearly see that the LSTM model fits well in predicting the time series data of PM_2.5_ concentration, but there are still shortcomings. It is evident that the low valley of PM_2.5_ concentration has a negative value, but theoretical knowledge shows that LSTM has a certain dependence when extracting features from long-term data. If the data volume is insufficient or the quality is not high, it may affect the performance of the model. Therefore, in the LSTM model, overfitting occurred, causing the prediction curve to maintain the prediction trend of the previous state without timely modification.

**Figure 9:**
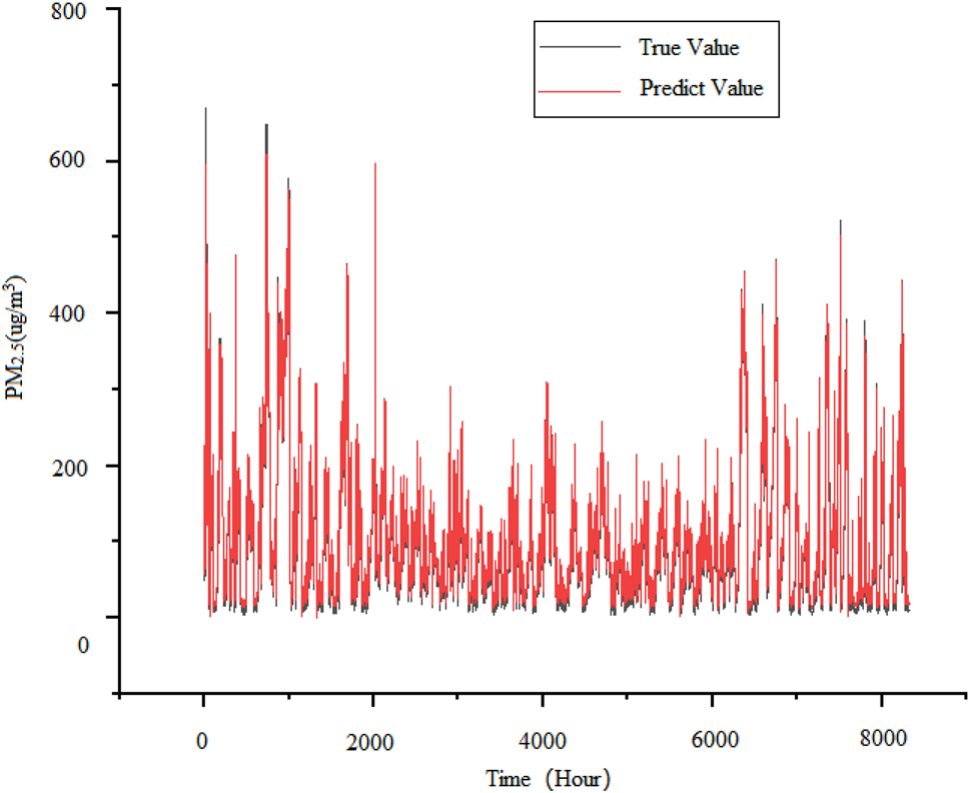
Predicted demerits of ALSTM model.

This paper developed an attention mechanism-based model ALSTM model so as to have a more intuitive understanding of its data requirements. The model enhances the key information of data features in the network by fully connecting the output of LSTM with the attention module, thereby improving the accuracy of PM_2.5_ concentration prediction. The prediction results are shown in Fig. 9. It can be clearly seen that after adding attention, the prediction model has a good fit at both peak and valley values, but there is still a gap from accurately predicting the trend of PM_2.5_ concentration. Furthermore, it can be observed in Fig. 9 that all of its valleys cannot be well predicted. However, for the overall prediction curve, the addition of attention mechanism has a promoting effect on the entire network model.

**Figure 10:**
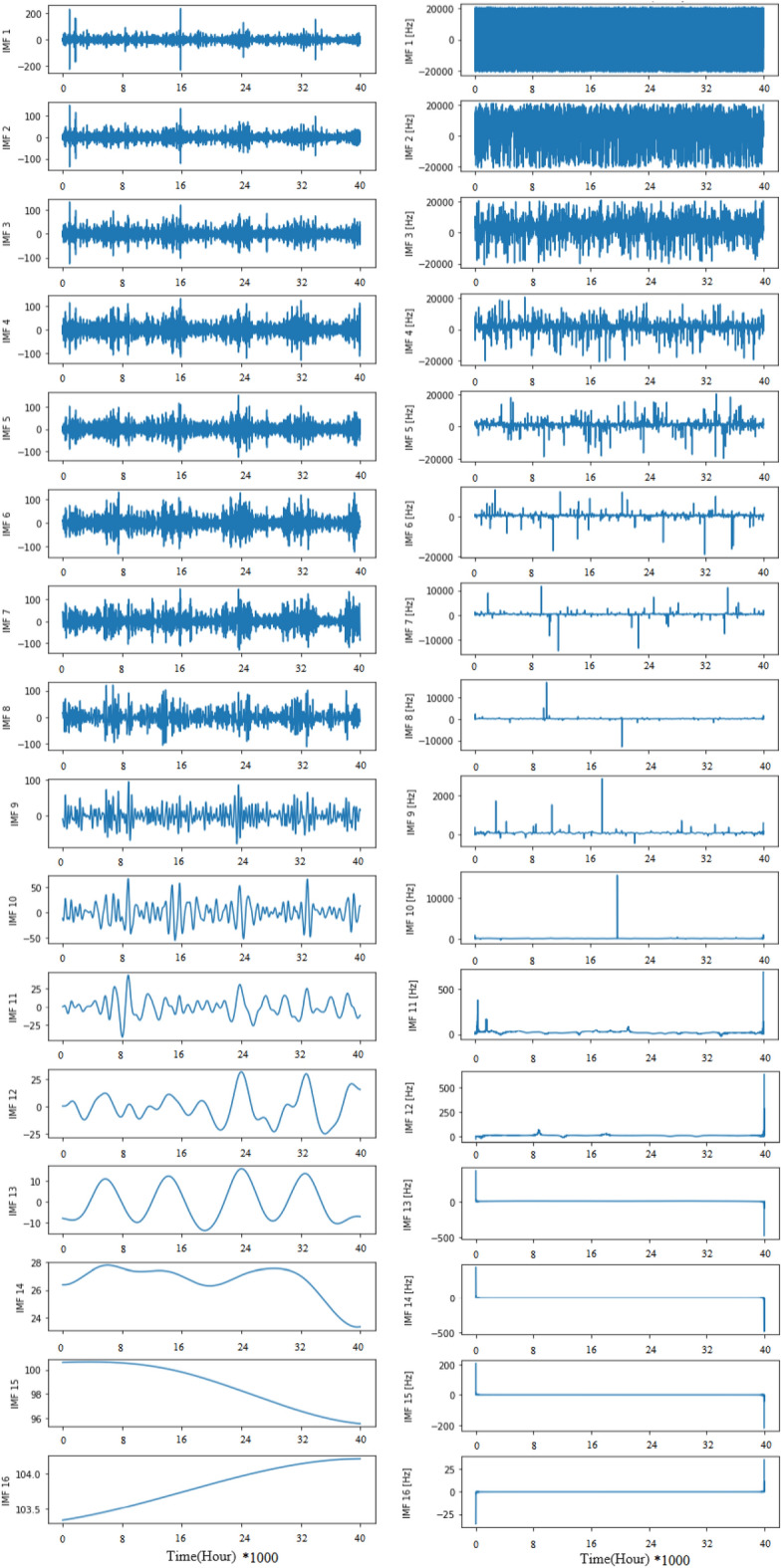
IMFs and instantaneous frequency after raw data decomposition.

**Figure 11:**
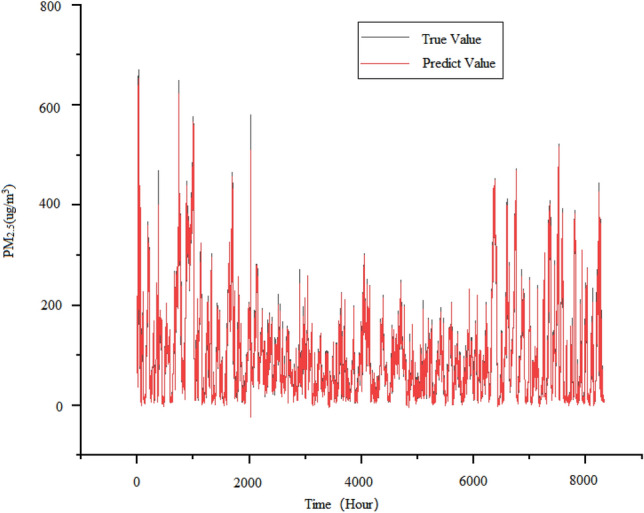
Predicted demerits of EEMD-LSTM model.

To make the comparative experiment more convincing, a set of experimental groups based on EEMD decomposition were used in the experiment. The decomposed sub sequences and instantaneous frequency^33^ graphs are shown in Fig. 10. Based on LSTM, we first use ensemble empirical mode decomposition to process the data. It decomposes the original signal and the instantaneous frame rate of each signal. Then, the decomposed modal signal is used to predict the time series of PM_2.5_ using the model, and finally, the predicted signals are combined to form an output. The prediction results of the EEMD-LSTM model are shown in Fig. 11.

**Figure 12:**
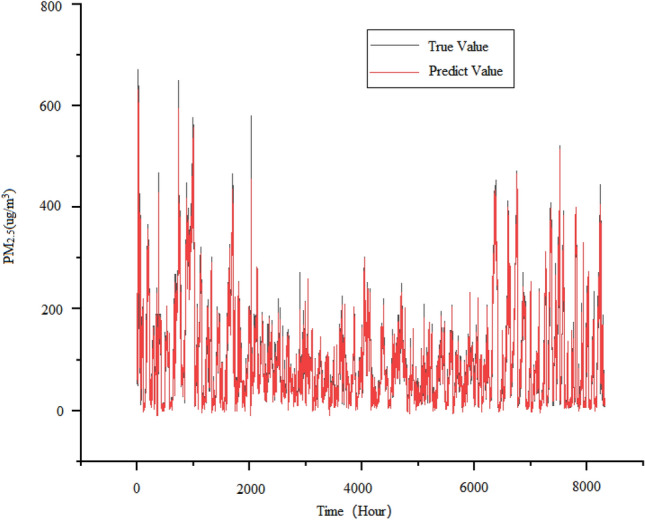
Predicted demerits of EEMD-ALSTM model.

The EEMD-ALSTM model also uses set empirical mode decomposition to process the data, and then predicts the decomposed modal signals using an attention-based LSTM model. Finally, as depicted in Fig. 12, the predicted signals are combined to form an output, and the prediction results of the EEMD-ALSTM model.

This work presents the predictions in the form of a table and evaluates the model using three basic indicators of predictive performance to have a better demonstration of the predictive performance of different models. The evaluation results are shown in Table 1.

**Table 1:** The evaluation results of each model.

Model	R^2^	MAE (μg/m^3^)	RMSE (μg/m^3^)
SVR	0.9249	12.6037	25.8623
LSTM	0.9316	13.0336	23.9177
ALSTM	0.9271	14.4295	24.6959
EEMD-LSTM	0.9487	0.8454	1.1384
EEMD-ALSTM	0.9550	0.6715	0.9451

The Table 1 could sensitively display that there are significant differences in the observed evaluation indicators under different model predictions. The R^2^ of the EEMD-ALSTM model is the highest, indicating that the model has the best regression stability. When predicting the air concentration of PM_2.5_, the prediction results are also the most reliable. In addition, the MAE and RMSE of this model are superior to other models, indicating that the EEMD-ALSTM model provides the most accurate prediction results when predicting PM_2.5_. In addition, based on the prediction results of ALSTM and EEMD-LSTM, the adding EEMD decomposition significantly improves the overall performance of the model in the experiment. Especially by reducing the MAE and RMSE by about 90%, the EEMD decomposition method has a significant effect in reducing the high nonlinearity of PM_2.5_ data. According to the experimental results of the EEMD-ALSTM and EEMD-LSTM model, it was found that while maintaining similar R_2_ correlation coefficients, the MSE and RMSE of the EEMD-ALSTM model decreased by about 15%, and the stability of the model in the prediction process was significantly improved. Above all, the increase in attention mechanism after simultaneously decomposing EEMD could improve the accuracy of the model in predicting PM_2.5_ concentration. In other words, the increase in attention enhances the models ability to extract features from data information and preserve and transmit as many important features as possible.

## Discussion

We plot and compare the predicted results of all models in this experiment using data from the test set, rather than comparing all PM_2.5_ concentration data. There are two reasons for using this method for drawing. The first is the training set has already been used once during model training, but if when conducting experimental predictions, the training set data is used again for drawing, which cannot fully reflect the advantages and disadvantages of the model; The second reason is that if all the data is plotted, the difference between the predicted and true values and the overall fit of the image cannot be reflected in the image.

Before using the data, the first processing of the data involves removing empty data points from the original dataset, making the original data a continuous time series. The advantage of this approach is that it adopts the dropout concept to prevent overfitting of the model. When cutting the entire data, use a 24-h PM_2.5_ concentration as a spline data. In normal production and life, people often pay more attention to the air environment quality of the next day. Therefore, the data from the previous 24 h is used to predict the data from the next 1 h.

In the comparison of the results between the LSTM and ALSTM model, we can find that the LSTM model has about 10% stronger error evaluation data than the ALSTM model. However, after EEMD decomposition and prediction, the ALSTM model is about one-third stronger than the LSTM models prediction data. This is because the network structure of the ALSTM model is complex, resulting in slightly worse performance than the LSTM model; However, after adding EEMD to decompose the data, the overall model data will reverse the LSTM model. This is because the changes in PM_2.5_ content in the air are highly nonlinear and therefore it is necessary to reduce the nonlinearity of data. In this way, when using the model, the feature information in the data can be more accurately extracted, thus it easier to make accurate predictions (Supplementary Information).

## Conclusions

Recently, many scholars in the field of environmental protection have become increasingly interested in predicting PM_2.5_ concentrations. With the continuous improvement of urban air pollution prediction and management, many cities have established air quality monitoring stations. How to effectively utilize the data collected by these monitoring stations to improve urban air quality has become an important issue. To address this issue, we propose an LSTM based on EEMD and attention mechanism for PM_2.5_ concentration prediction.

Summarizing the results of this work, we elicit the main conclusions as follows.

1. For the sequence of PM_2.5_ concentration with high nonlinearity, how to handle the highly nonlinear the sequence is inevitable. This work first uses the EEMD to process the sequence, in order to effectively improve its nonlinearity and form regular sequence in their respective modes.
2. This article proposes using an attention mechanism model to optimize the prediction of LSTM, which has better performance compared to traditional machine learning models and separately predicted models. The reason is that the attention mechanism can more accurately extract the correlation of information in signals and assign greater weights with emphasis.
3. This article conducted 24-h predictions for each model, and the prediction results showed that the EEMD-ALSTM model had better predictive performance. MAE, RMSE and R^2^ were 0.6715 µg/m^3^, 0.9451 µg/m^3^ and 0.9550 respectively.
4. However, when designing network parameters, we used fixed network parameters, which may not necessarily be the best choice for the current network model. The selection of network parameters has a significant impact on the performance and stability of the model. Therefore, in future research, we will consider introducing network parameter lookup methods to further improve the capabilities of the EEMD-ALSTM model. The network parameter search method can automatically adjust network parameters based on the characteristics of data and prediction needs to obtain the best prediction results. This will enable the model to better adapt to different data features and prediction tasks, thereby improving the accuracy and stability of prediction.

In summary, the EEMD-ALSTM prediction model based on EEMD, attention mechanism and LSTM to predict PM_2.5_ concentration. The experimental results show that it performs well in terms of predictive ability with high accuracy and stability. Future research will further optimize the network parameters of the model to improve its predictive ability and provide more reliable decision-making basis for environmental management and public health.

### Supplementary Information

Supplementary Information.

## Data Availability

Data is provided within the manuscript or supplementary information files.

## References

[1] Bai KX, Li K, Chang NB, Gao W (2019). Advancing the prediction accuracy of satellite-based PM_2.5_ concentration mapping: A perspective of data mining through in situ PM_2.5_ measurements. Environ. Pollut..

[2] Zhang DY, Bai KX, Zhou YY, Shi RH, Ren HY (2019). Estimating ground-level concentrations of multiple air pollutants and their health impacts in the Huaihe River Basin in China. Int. J. Env. Res. Pub. He..

[3] Byun D, Schere KL (2006). Review of the governing equations computational algorithms and other components of the models-3 community multiscale air quality (CMAQ) modeling system. Appl. Mech. Rev..

[4] Jin Q, Fang XY, Wen B, Shan AD (2017). Spatio-temporal variations of PM_2.5_ emission in China from 2005 to 2014. Chemosphere.

[5] Reichstein M (2019). Deep learning and process understanding for data-driven earth system science. Nature.

[6] Zhou HY, Zhang F, Du ZJ, Liu RY (2021). Forecasting PM_2.5_ using hybrid graph convolution-based model considering dynamic wind-field to offer the benefit of spatial interpretability. Environ. Pollut..

[7] LeCun Y, Bengio Y, Hinton G (2015). Deep learning. Nature.

[8] Liu YD (2023). ynamics evolution prediction from time series data with recurrent neural networks in a complex system. Int. J. Mod. Phys. C.

[9] Elman JL (1990). Finding structure in time. Cognitive Sci..

[10] Hochreiter S, Schmidhuber J (1997). Long short-term memory. Neural Comput..

[11] Wang WL, Mao WJ, Tong XL, Xu G (2021). A novel recursive model based on a convolutional long short-term memory neural network for air pollution prediction. Remote Sens. Basel.

[12] Wang SY, Zhang SB, Huang XP, Chang LB (2023). A high-efficiency spaceborne processor for hybrid neural networks. Neurocomputing.

[13] Ma H, Liang SL (2022). Development of the GLASS 250-m leaf area index product (version 6) from MODIS data using the bidirectional LSTM deep learning model. Remote Sens. Environ..

[14] Parsaeimehr E, Fartash M, Torkestani JA (2023). Improving feature extraction using a hybrid of CNN and LSTM for entity identification. Neural Process. Lett..

[15] Niu ZY, Zhong GQ, Yu H (2021). A review on the attention mechanism of deep learning. Neurocomputing.

[16] Huang GY, Li XY, Zhang B, Ren JD (2021). PM_2.5_ concentration forecasting at surface monitoring sites using GRU neural network based on empirical mode decomposition. Sci. Total Environ..

[17] Yan X, Zuo C, Li ZQ, Chen HW, Jiang YZ, He B, Liu HM, Chen JY, Shi WZ (2023). Cooperative simultaneous inversion of satellite-based real-time PM_2.5_ and ozone levels using an improved deep learning model with attention mechanism. Environ. Pollut..

[18] Shadi A, Jamil A (2016). Assessing the accuracy of ANFIS, EEMD-GRNN, PCR, and MLR models in predicting PM_2.5_. Atmos. Environ..

[19] Yang H, Zhao JL, Li GH (2022). A new hybrid prediction model of PM_2.5_ concentration based on secondary decomposition and optimized extreme learning machine. Environ. Sci. Pollut. R..

[20] Yang H, Wang WQ, Li GH (2023). Prediction method of PM_2.5_ concentration based on decomposition and integration. Measurement.

[21] Zhu JQ, Deng F, Zhao JC, Zheng H (2021). Attention-based parallel networks (APNet) for PM_2.5_ spatiotemporal prediction. Sci. Total Environ..

[22] Al-Janabi S, Alkaim A, Al-Janabi E, Aljeboree A, Mustafa M (2021). Intelligent forecaster of concentrations (PM_2.5_, PM_10_, NO_2_, CO, O_3_, SO_2_) caused air pollution (IFCsAP). Neural Comput. Appl..

[23] Usmani RSA, Pillai TR, Hashem IAT, Marjani M, Shaharudin RB, Latif MT (2023). Artificial intelligence techniques for predicting cardiorespiratory mortality caused by air pollution. Int. J. Environ. Sci. Te..

[24] Huang NE, Shen Z, Long SR, Wu MC, Shih HH, Zheng Q, Yen NC, Tung CC, Liu HH (1998). The empirical mode decomposition and the Hilbert spectrum for nonlinear and non-stationary time series analysis. Proc. R. Soc. Lond. A.

[25] Wu Z, Huang NE (2004). A study of the characteristics of white noise using the empirical mode decomposition method. Proc. R. Soc. Lond. A.

[26] Gupta M, Wadhvani R, Rasool A (2023). A real-time adaptive model for bearing fault classification and remaining useful life estimation using deep neural network. Knowl. Based Syst..

[27] Harichandran A, Raphael B, Mukherjee A (2023). Equipment activity recognition and early fault detection in automated construction through a hybrid machine learning framework. Comput. Aided Civ. Inf..

[28] Li B, Liu FY, Song QK, Zhang DP, Qiu HJ (2023). State estimation of complex-valued neural networks with leakage delay: A dynamic event-triggered approach. Neurocomputing.

[29] Almutairi MS, Almutairi K, Chiroma H (2023). Hybrid of deep recurrent network and long short term memory for rear-end collision detection in fog based internet of vehicles. Expert Syst. Appl..

[30] Camastra F, Capone V, Ciaramella A, Riccio A, Staiano A (2022). Prediction of environmental missing data time series by support vector machine regression and correlation dimension estimation. Environ. Modell. Softw..

[31] Guo L, Fang WG, Zhao QH, Wang X (2021). The hybrid prophet-svr approach for forecasting product time series demand with seasonality. Comput. Ind. Eng..

[32] Thissen U, Van Brakel R, De Weijer AP, Melssen WJ, Buydens LMC (2003). Using support vector machines for time series prediction. Chemometr. Intell. Lab. Syst..

[33] Lozano M, Fiz JA, Jané R (2015). Automatic differentiation of normal and continuous adventitious respiratory sounds using ensemble empirical mode decomposition and instantaneous frequency. IEEE J. Biomed. Health.

